# Identification of autoreactive B cells with labeled nucleosomes

**DOI:** 10.1038/s41598-017-00664-0

**Published:** 2017-04-04

**Authors:** Vincent Gies, Alain Wagner, Cécile Seifert, Aurélien Guffroy, Jean-D. Fauny, Anne-M. Knapp, Jean-L. Pasquali, Thierry Martin, Hélène Dumortier, Anne-S. Korganow, Pauline Soulas-Sprauel

**Affiliations:** 1 CNRS UPR 3572 “Immunopathology and Therapeutic Chemistry”/Laboratory of Excellence Medalis, Institute of Molecular and Cellular Biology (IBMC), Strasbourg, France; 2 Department of Clinical Immunology and Internal Medicine, National Reference Center for Autoimmune Diseases, Hôpitaux Universitaires de Strasbourg, Strasbourg, France; 3 Laboratory of Functional ChemoSystems, CNRS-University of Strasbourg UMR 7199/Laboratory of Excellence MEDALIS, Faculté de Pharmacie, Université de Strasbourg, 74 route du Rhin, 67400 Illkirch, France; 4 UFR Médecine, Université de Strasbourg, Strasbourg, France; 5 UFR Sciences pharmaceutiques, Université de Strasbourg, Illkirch-Graffenstaden, France

## Abstract

The pathogenesis of autoimmune diseases has not been completely elucidated yet, and only a few specific treatments have been developed so far. In autoimmune diseases mediated by pathogenic autoantibodies, such as systemic lupus erythematosus, the specific detection and analysis of autoreactive B cells is crucial for a better understanding of the physiopathology. Biological characterization of these cells may help to define new therapeutic targets. Very few techniques allowing the precise detection of autoreactive B cells have been described so far. Herein we propose a new flow cytometry technique for specific detection of anti-nucleosome B cells, which secrete autoantibodies in systemic lupus erythematosus, using labeled nucleosomes. We produced different fluorochrome-labeled nucleosomes, characterized them, and finally tested them in flow cytometry. Nucleosomes labeled via the cysteines present in H3 histone specifically bind to autoreactive B cells in the anti-DNA transgenic B6.56R mice model. The present work validates the use of fluorochrome-labeled nucleosomes via cysteines to identify anti-nucleosome B cells and offers new opportunities for the description of autoreactive B cell phenotype.

## Introduction

Many autoimmune diseases, such as systemic lupus erythematosus (SLE), are characterized by the presence of B cells that are directed against self antigens (i.e. autoreactive B cells) and produce autoantibodies (autoAbs)^1^. In these autoimmune diseases mediated by pathogenic autoAbs, the specific detection and analysis of autoreactive B cells is a key point to understand the physiopathology of the disease. The phenotypic analysis of these cells by flow cytometry would potentially lead to the description of new specific markers of autoreactive B cells. In addition it could give interesting information about the biological abnormalities which characterize these cells, and may help to find new therapeutic targets.

In healthy individuals, tolerance mechanisms prevent the development and the activation of autoreactive B cells, but these mechanisms are deficient in autoimmune diseases. Indeed SLE – a prototypic autoantibody-mediated autoimmune disease – is characterized by a loss of tolerance to nuclear antigens, due to a deficient clearance of apoptotic cells^2, 3^. Nuclear antigen recognition leads to an abnormal auto-reactive immune response, in which B cells play a central role with the production of pathogenic autoAbs, as anti-double stranded DNA (anti-dsDNA) or anti-nucleosome antibodies^4–7^. Anti-nucleosome antibodies are part of a large family of antibodies directed against epitopes of histones, dsDNA or conformational epitopes created by the interactions between dsDNA and histones^8^. They may precede the clinical development of SLE up to 10 years^4^, and as anti-DNA antibodies, they are SLE-specific and associated with the disease activity^9^. These autoantibodies form immune complexes within blood vessels and kidneys leading to chronic inflammation, and thus play a critical role in the pathogenesis^6, 10–12^. However the exact phenotype of B cells producing these autoAbs in SLE remains unknown.

Very few techniques allowing the detection of antigen-specific autoreactive B cells using flow cytometry have been described in the literature^13–18^. In SLE, some studies used small linear peptide sequences^14, 16^, limiting the number of autoepitopes (protein sequences recognized by autoreactive B cells) and therefore resulting in the isolation of only a small fraction of the pathogenic autoreactive B cells. In addition, other studies used an anti-idiotype antibody called 9G4 to label and characterize autoreactive B cells from SLE patients^15, 19–21^. However 9G4 recognizes B cell antigen receptors (BCRs) on many autoreactive B cells, and also on other unrelated targets that are not linked to the pathogenesis of the disease, such as N-acetyl-lactosamine determinants of blood group antigens or CD45 surface protein^22–24^, limiting results interpretation. In order to develop a technique for the detection of autoreactive B cells by flow cytometry in SLE, we chose the nucleosome – the basic unit of chromatin – as an autoantigen. Nucleosome is composed of 146 DNA base pairs wrapped around two copies of histones H2A, H2B, H3 and H4 (the core histones)^25, 26^. Free circulating DNA is usually not found in SLE patient, but rather exists in the form of circulating nucleosomes^27^, suggesting that the nucleosome is both the driving immunogen and the target of anti-dsDNA antibodies. The nucleosome, the major autoantigen in SLE^28–30^, possesses multiple autoepitopes, including DNA. Therefore, the use of labeled nucleosomes could be more adapted to the isolation of a large spectrum of representative pathogenic B cells than the use of a linear peptide that can only isolate a small fraction of autoreactive B cells.

The aim of this study was to produce and characterize fluorochrome-labeled nucleosomes, and finally test them for the detection of anti-nucleosome B cells. Cysteine-labeled nucleosomes display a suitable fluorescence and can specifically bind to autoreactive B cells in the anti-DNA transgenic B6.56R mice model. In addition, the use of antibodies blocking the BCR inhibited this labeling, arguing for a BCR selective binding of the labeled nucleosomes. Thus, the present work opens up the use of cysteine labeled nucleosomes to identify and characterize anti-nucleosome B cells for a better understanding of SLE physiopathology.

## Results and Discussion

### Nucleosome labeling and characterization

**Figure 1:**
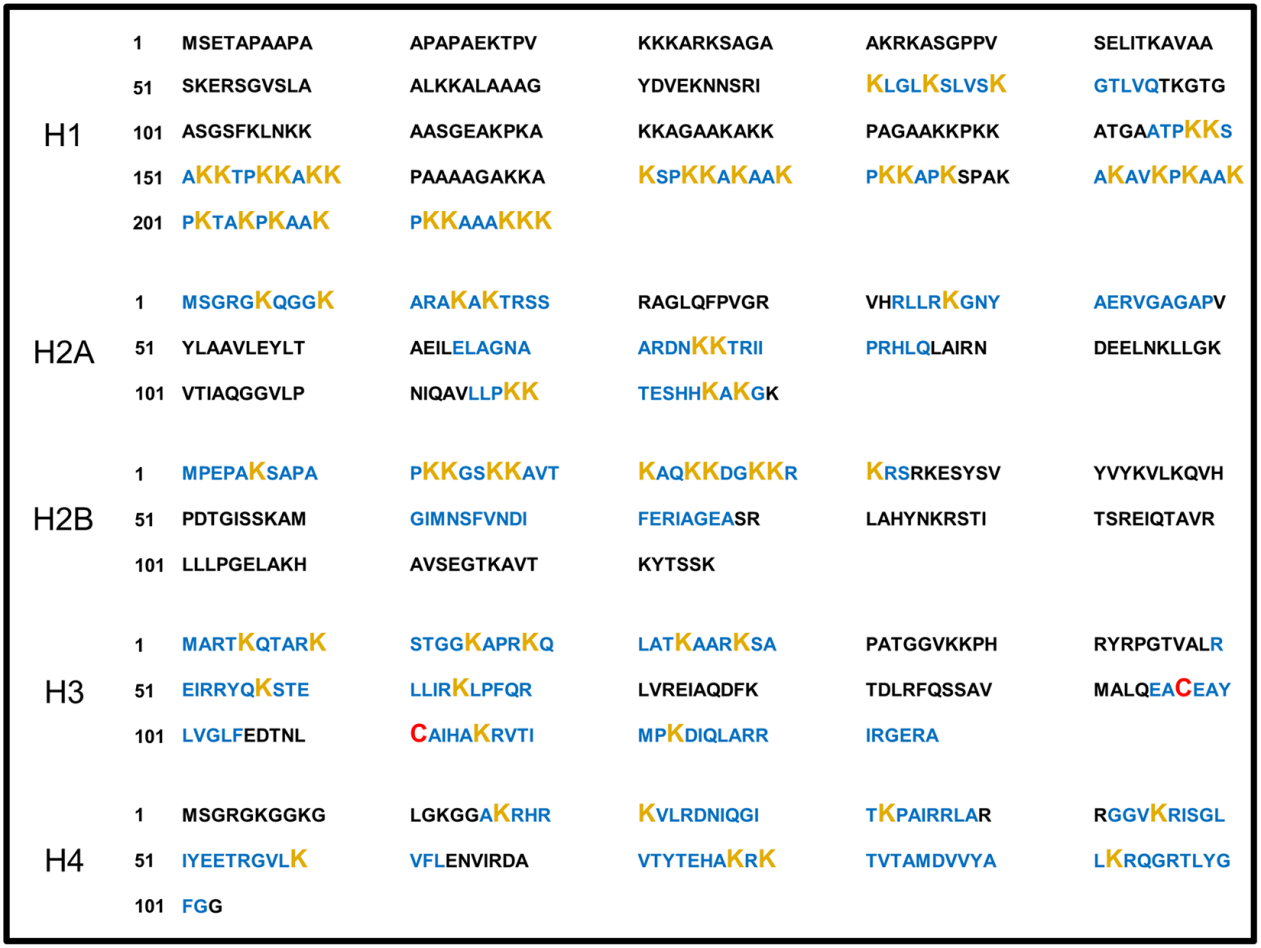
Protein sequences of histones. Protein sequences of histone regions which are exposed at the surface (*blue*). Lysine bearing free amine group (*orange*); Cysteine (*Red*).

In order to develop a new flow cytometric method to detect autoreactive B cells, we chose the nucleosome as an autoantigen. Nucleosomes were isolated from L1210 or HEK293 cell line (referred as “native nucleosomes”) and labeled. As free amino groups are abundant at the major epitope sites of nucleosomes (Fig. 1), strategies using lysine labeling were excluded, to avoid a blocking of major epitopes and a potential loss of recognition by autoreactive B cells. We performed a cysteine-labeling of the histone H3 – the only one among the core histones to possess this amino-acid – with a AlexaFluor 488 maleimide (referred as “cysteine nucleosomes”) (Fig. 1). High salt concentration was used to increase thiols availability to the maleimide^31, 32^, followed by a desalting dialysis to allow gradual reassembly of the nucleosome^33, 34^.

**Figure 2:**
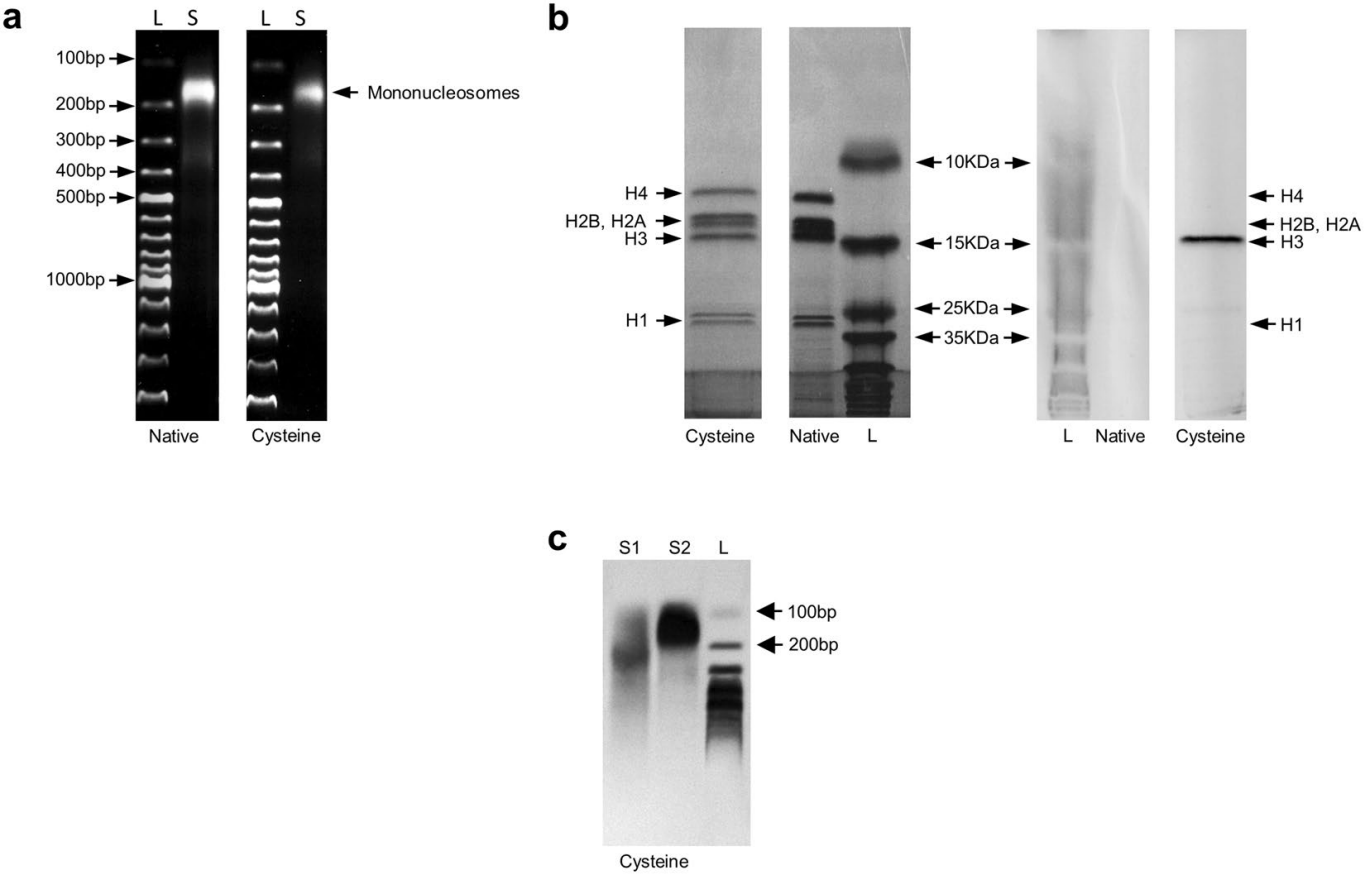
Characterization of native and labeled nucleosomes (cropped gel). (**a**) Analysis of nucleosome DNA content (500 ng of DNA) by agarose gel (1.2%) after proteinase K digestion. L: ladder; S: sample. (**b**) Analysis of nucleosome proteins (equivalent to 1.5 µg of DNA) by SDS/PAGE (18%) after Coomassie Blue staining (*left*) or under UV light before Coomassie Blue staining (*right*). Native: native nucleosome; Cysteine: cysteine nucleosome. (**c**) Analysis of cysteine nucleosome DNA content (500 ng of DNA) by agarose gel (1.2%) before (S1) or after (S2) proteinase K digestion. L: ladder; S: sample. The non-cropped images of the gels are presented in Supplementary Fig. S3.

Samples were extensively dialyzed after nucleosome isolation and labeling, to ensure the removal of small proteins and free reagent (Supplementary Fig. S1). DNA analysis on 1.2% agarose gel, after proteinase K digestion, showed the presence of a 150 bp band, corresponding to mononucleosomes (Fig. 2a). Separation and characterization of proteins – based on polyacrylamide gel electrophoresis – highlighted the presence of the histone octamer, composed of the four histones H2A, H2B, H3, H4, and histone H1, which is a linker between the histones octamer and the DNA^35^. The purity of the samples was high considering the almost complete absence of other bands (Fig. 2b, left). Exposition of the polyacrylamide gel to UltraViolet, before Coomassie Blue staining, allowed the detection of the AlexaFluor 488 in the nucleosomes. The labeling was specific to H3, the only histone containing cysteines. Labeled H3 displayed a strong fluorescence (Fig. 2b, right) that was confirmed by flow cytometry analysis (Supplementary Fig. S2). In addition, the integrity of cysteine nucleosome was evaluated by separation on 1.2% agarose gel. Cysteine nucleosomes ran much slower than the DNA they contain, which migrated to 150 bp after proteinase K digestion^36–38^, confirming that cysteine nucleosomes were intact (Fig. 2c).

### Effective nucleosome-autoAbs interaction after nucleosome labeling

**Figure 3:**
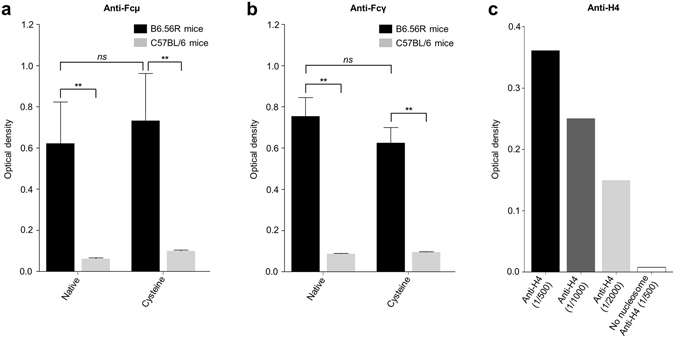
Recognition of labeled nucleosomes by antibodies from B6.56R and control (C57BL/6) mice. Serum-derived IgM (**a**) or IgG (**b**) from B6.56R mice (*black*; n = 5) and C57BL/6 mice (*grey*; n = 5) were tested by indirect ELISA against nucleosomes. The histograms represent the optical density (mean ± SEM) obtained with labeled or native nucleosomes. (**c**) The reactivity of an anti-H4 antibody (1/500, 1/1000 and 1/2000 dilutions) was tested by indirect ELISA against cysteine nucleosomes. A negative control without nucleosome was added (white bar). Statistical comparison was carried out using nonparametric two-tailed Mann-Whitney test. Native: native nucleosome; Cysteine: cysteine nucleosome. **P < 0.005.

An ELISA technique was used to test whether anti-nucleosome autoAbs recognized such labeled nucleosomes. A less effective antigen-antibody interaction could occur if the labeling leads to nonaccessible epitopes or a conformational change of the histone octamer. Plates were coated with native or cysteine nucleosomes, then incubated with serum containing anti-nucleosome IgG and IgM. For this purpose, we used sera from B6.56R mice (56R mice on C57BL/6 background), i.e. transgenic mice whose B cells express autoreactive BCRs and produce autoAbs directed against dsDNA, ssDNA and histones/DNA complexes^39, 40^. Therefore, serum-derived antibodies from B6.56R mice recognize largely DNA and their ability, through that recognition, to bind nucleosomes was compared to serum-derived antibodies from control C57BL/6 mice. Native nucleosomes were highly recognized by IgM (Fig. 3a) or IgG (Fig. 3b) autoAbs from B6.56R mice, unlike immuglobulins from control mice. As expected, the labeling of nucleosomes did not change the binding capacity of IgM (Fig. 3a) or IgG (Fig. 3b) from B6.56 R. To assess the antigenic composition and how these labeled nucleosomes would react with autoantibodies against histones, we used an anti-H4 antibody specific for the amino-terminal sequence of histone H4 – a surface exposed region (Fig. 1). The anti-H4 antibody reacted with labeled nucleosomes, in a dose-dependent manner (Fig. 3c). Therefore, cysteine nucleosomes appeared as good candidates for a flow cytometry use.

### Detection of anti-nucleosome B cells by flow cytometry using labeled nucleosomes

**Figure 4:**
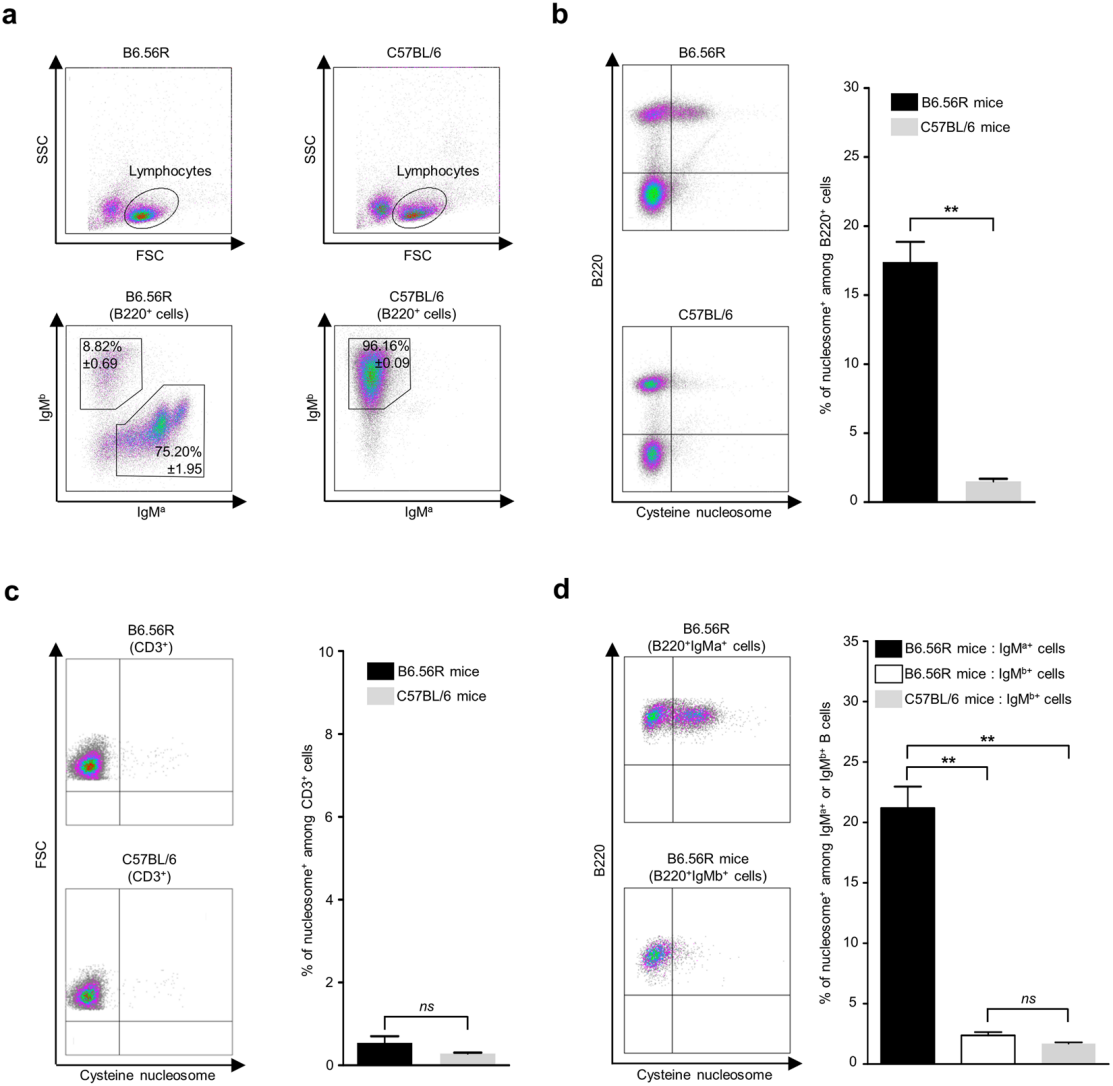
Cysteine nucleosome selectively stained the B cells carrying the transgenic heavy chain, from B6.56R mice. (**a**) Plots of cysteine nucleosomes loaded lymphocytes (upper diagrams) and flow cytometry analysis of IgM^a+^ or IgM^b+^ splenic B cells subsets (lower diagrams; percentage of B220^+^ cells in the lymphocyte gate) in B6.56R mice compared to C57BL/6 mice. (**b**, *left*) Representative plots of splenic B220^+^ B cells staining with labeled nucleosomes, in B6.56R mice and C57BL/6 mice. (**b**, *right*) Frequency of splenic cysteine nucleosome positive B cells (percentage among B220^+^ cells) in B6.56R mice (*black*) and C57BL/6 mice (*grey*). (**c**, *left*) Representative plots of CD3^+^ T cells staining with cysteine nucleosomes, in B6.56R mice and C57BL/6 mice. (**c**, *right*) Frequency of splenic cysteine nucleosome positive T cells (percentage among CD3^+^ cells) in B6.56R mice (*black*) and C57BL/6 mice (*grey*). (**d**, *left*) Representative plots of splenic IgM^b+^B220^+^ or IgM^a+^B220^+^ B cells staining with labeled nucleosomes, in B6.56R mice. (**d**, *right*) Frequency of cysteine nucleosome positive B cells (percentage among IgM^b+^B220^+^ or IgM^a+^B220^+^ cells) in B6.56R mice (*black*: *IgM* ^*a*^; *white*: *IgM* ^*b*^) compared to IgM^b+^B220^+^ cells from C57BL/6 mice (*grey*). Mean ± SEM for 5 mice. Statistical comparison was carried out using nonparametric two-tailed Mann-Whitney test. **P < 0.005.

The autoreactive 56R heavy chain knock-in mice model is a very useful and well-described model in order to study how tolerance against an ubiquitous antigen takes place^41, 42^. When combined with endogenous light chain, the 56R transgene – a mutated form of the anti-DNA 3H9 transgenic heavy chain – forms a BCR with increased affinity and specificity for dsDNA, ssDNA and histones/DNA complexes^39, 40^. The detection of autoreactive B cells in this model is based on the identification of cells carrying the transgene by PCR, or by flow cytometry using anti-haplotype antibodies: anti-IgM^a^ antibody binds to BCR expressing the “a” haplotype, which corresponds to the 56R transgenic heavy chain that potentially recognizes the autoantigens, and anti-IgM^b^ antibody binds to BCR expressing the “b” haplotype, i.e. the endogenous heavy chains^42^. As shown in Fig. 4a, most B cells in B6.56R mice expressed the transgene, with a majority of B220^+^ B cell expressing IgM^a^ (75.20% ± 1.95) compared to IgM^b^ (8.82% ± 0.69), while C57BL/6 mice did not express the transgenic allele and therefore had only IgM^b^ B cells (96.16% ± 0.09).

In B6.56R mice, B cells expressing the 56R transgene partially escape tolerance mechanisms and produce autoAbs in the periphery^43–45^. However, due to editing of the variable regions or to the possibility to pair with particular light chains, only a fraction (36%) of spontaneous hybridomas from B6.56R mice really bind the autoantigen^46^. Consequently the identification of the heavy chain using only anti-IgM^a^ labeling is not the best way to determine the phenotype of autoreactive B cells. The co-staining with labeled nucleosomes could, however, resolve this issue.

Indeed, a significant proportion of B cells from B6.56R mice recognized cysteine nucleosomes, while only a few B cells from C57BL/6 mice were labeled (17.40% ± 1.46 vs. 1.51% ± 0.19) (Fig. 4b). In contrast, T cells did not bind cysteine nucleosomes (Fig. 4c) both in B6.56R mice (0.54% ± 0.16) and in C57BL/6 mice (0.28% ± 0.02). Microscopy analysis confirmed the absence of nucleosomes’ labeling on B cells from control mice and a nucleosome punctuate membrane staining on a fraction of B cells in B6.56R mice (Supplementary Fig. S4a,b). In addition, this staining was highly specific to IgM^a+^ B cells, with only few IgM^b+^ B cells being labeled in B6.56R mice (21.19% ± 1.78 in IgM^a+^ B cell population, vs. 2.38% ± 0.27 in IgM^b+^ B cell population) (Fig. 4d), confirming that the labeling was selective of transgenic IgM^a+^ B cells.

**Figure 5:**
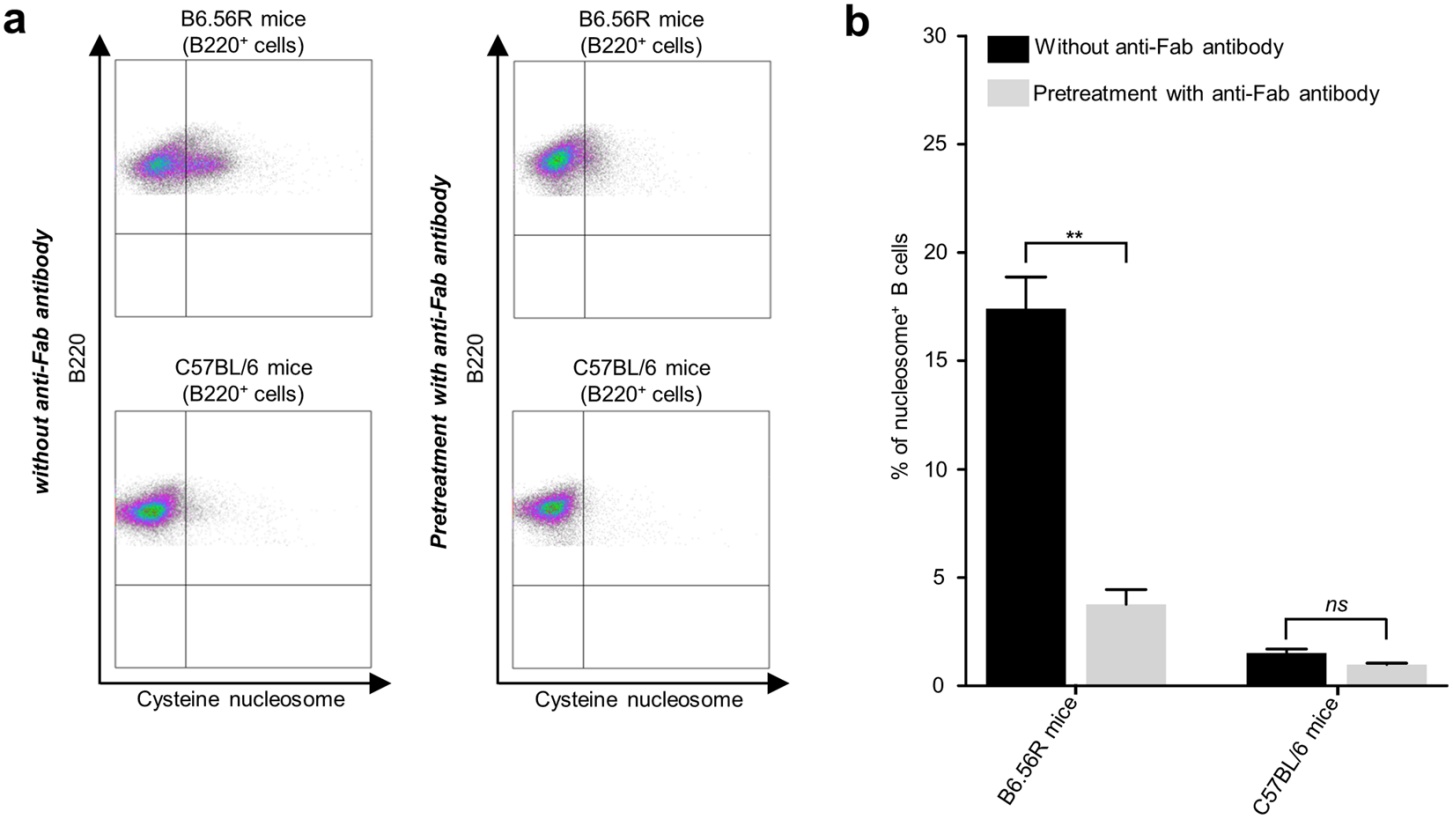
Cysteine nucleosome staining on IgM^a+^ B cells from B6.56R mice is BCR-specific. (**a**) Representative plots of splenic B220^+^ cells staining with labeled nucleosomes, without (*left*) or with (*right*) pretreatment with an anti-Fab antibody, in B6.56R mice compared to C57BL/6 mice. (**b**) Frequency of cysteine nucleosome^+^ B220^+^ cells (percentage among B220^+^ cells; mean ± SEM; n = 5), with (*grey*) or without (*black*) pretreatment with an anti-Fab antibody, in B6.56R mice compared to C57BL/6 mice. Statistical comparison was carried out using nonparametric two-tailed Mann-Whitney test. **P < 0.005.

To demonstrate that the staining with nucleosomes on IgM^a+^ B cells was BCR-specific, B cells were incubated with an anti-mouse Fab antibody before labeling with nucleosomes. This antibody binds to the BCR (containing two Fab fragments), leading to steric hindrance^47^ and partial BCR internalization^48, 49^, and thereby prevents antigen recognition. Pre-incubation with an anti-Fab antibody highly inhibited nucleosome binding on B cells from B6.56R mice (17.40% ± 1.46 without anti-Fab vs. 3.76% ± 0.68 with anti-Fab antibodies). In contrast, we did not detect any significant inhibition on B cells from C57BL/6 mice (1.51% ± 0.19 without anti-Fab vs 0.98% ± 0.07 with anti-Fab antibodies) (Fig. 5). In addition, BCR clusters containing nucleosomes could be identified after B cell activation (Supplementary Fig. S4c). Together, these data confirmed that cysteine nucleosomes bind to IgM^a+^ B cells in a BCR specific manner.

**Figure 6:**
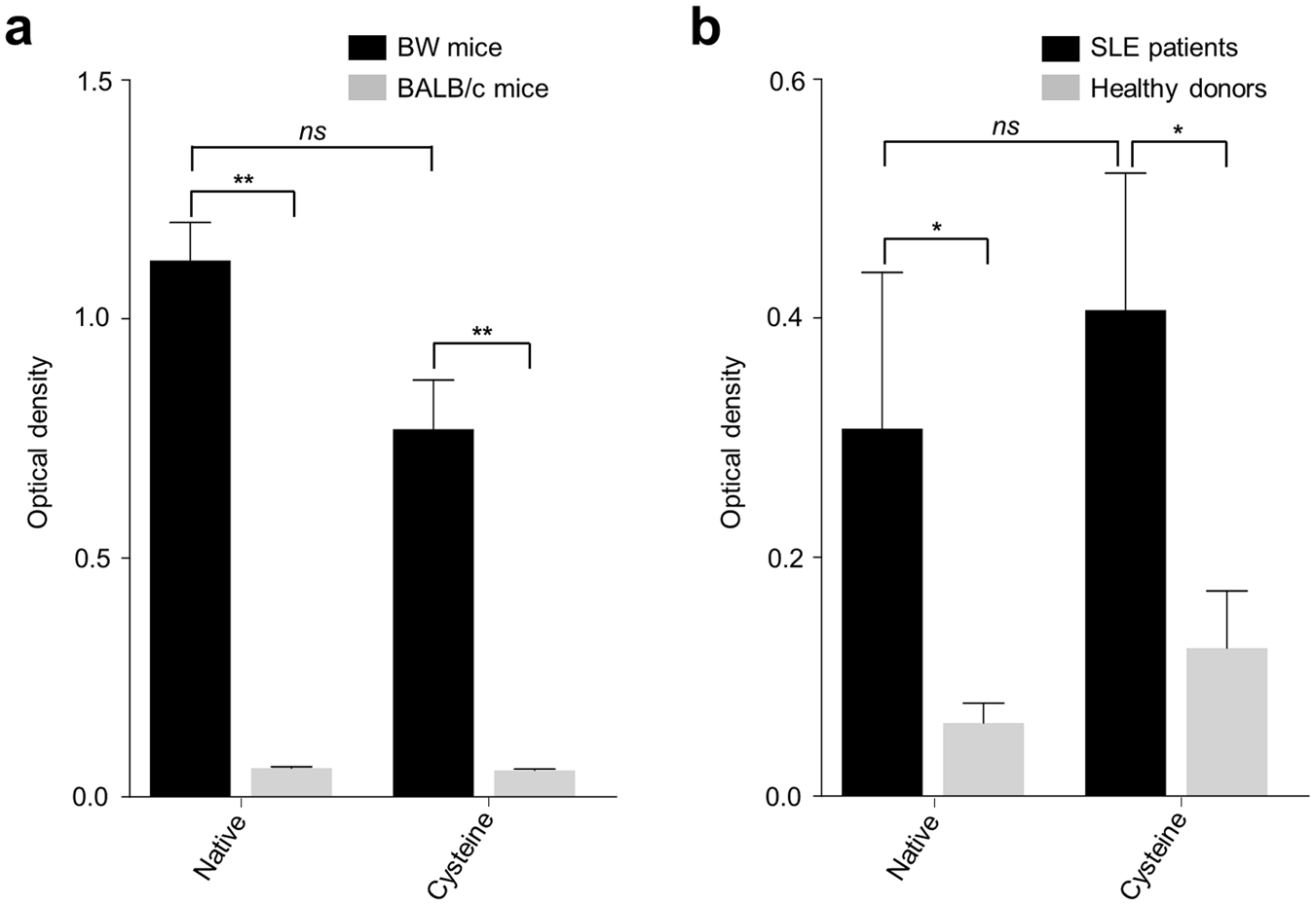
Recognition of labeled nucleosomes by antibodies from BW mice, BALB/c mice, SLE patients and healthy donors. (**a**) IgG from BW mice (*black*; n = 5) and BALB/c mice (*grey*; n = 5) sera were tested against nucleosomes in an indirect ELISA test. Nucleosomes were isolated from L1210 murine cell line (**b**) IgG from SLE patients (*black*; n = 5) and healthy donors (*grey*; n = 5) sera were tested against nucleosomes in an indirect ELISA test. Nucleosomes were isolated from HEK293 human cell line (**b**). Statistical comparison was carried out using nonparametric two-tailed Mann-Whitney test. Native: native nucleosome; Cysteine: cysteine nucleosome. *P < 0.05, **P < 0.005.

**Figure 7:**
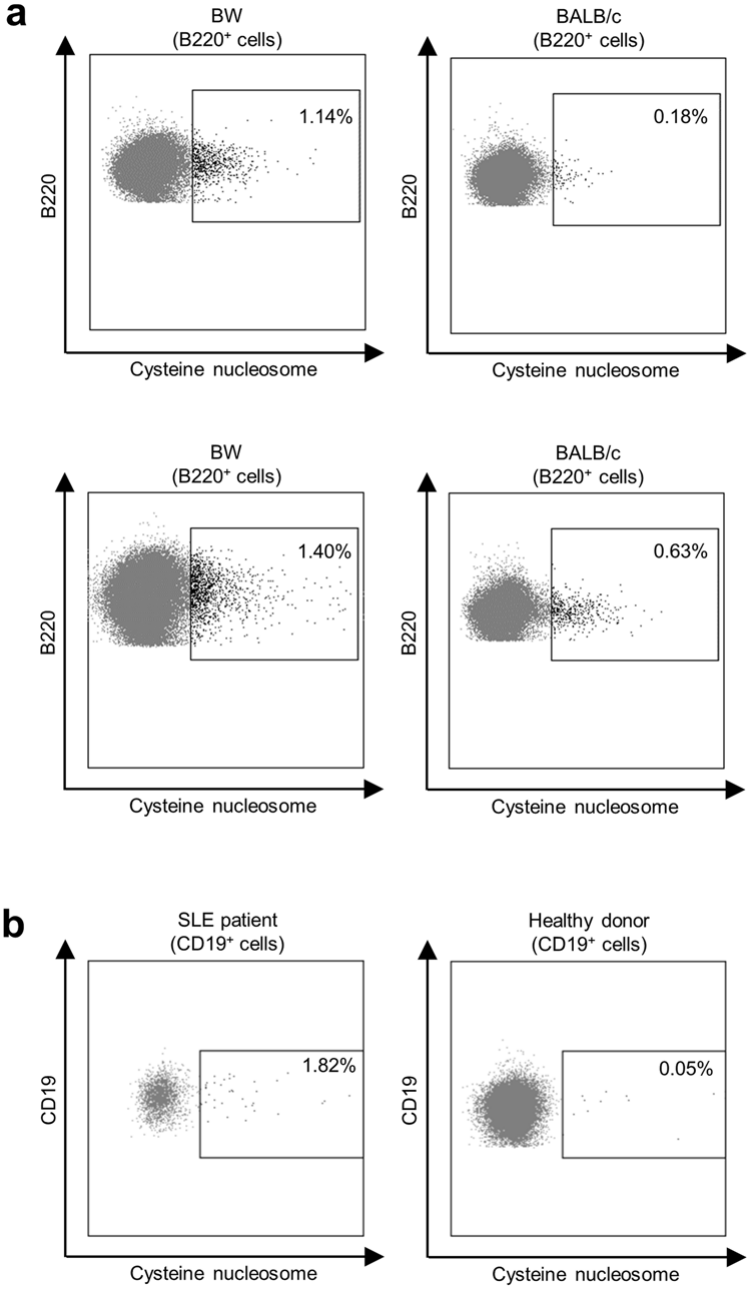
(**a**) Plots of nucleosome staining in splenic B220^+^ B cells in BW mice compared to BALB/c mice. (**b**) Plots of nucleosome staining in CD19^+^ B cells from SLE patient and healthy donor.

To extend our results, we performed an ELISA test using sera from BW mice, a mouse strain that spontaneously develop lupus-like disease^50^, and BALB/c mice as control. As expected, only BW mice sera reacted with native or cysteine nucleosomes. Similar results were obtained with serum from SLE patients and nucleosomes extracted from HEK293 cells, a human cell line (Fig. 6). A preliminary flow cytometry experiment on B cells from BW mice and one SLE patient showed a detectable staining with cysteine nucleosomes (Fig. 7). Altogether, these data showed that cysteine labeled nucleosome could be used to explore the phenotype of anti-nucleosome autoreactive B cells.

## Conclusion

The development of autoreactive B cells is tightly regulated in healthy individuals. A breakdown in tolerance mechanisms may result in the emergence of autoreactive B cells and the production of pathogenic autoAbs, leading to autoimmune disease development. In order to better characterize autoreactive B cells in SLE – a prototypic autoantibody-mediated autoimmune disease – we chose the main autoantigen responsible for pathogenic autoantibody production: the nucleosome. Different strategies were tested to set up a flow cytometry labeling protocol of autoreactive B cells with fluorescent nucleosomes. (i) One approach used nucleosomes labeled via EdU (5-ethynyl-2′-deoxyuridine), a nucleoside analog of thymidine, that is incorporated into DNA during active DNA synthesis^51^ and detected with a click reaction (“Huisgen’s reaction” or 1–3 dipolar cycloaddition)^52^. As copper, which is known to be toxic and cause protein alterations and DNA cleavages^53, 54^, is required for the click reaction to proceed, we stop using this method (data not shown). (ii) As a second approach, we produced nucleosomes coupled to the green fluorescent protein (GFP) by transducing HEK293T cells with a lentivirus allowing the expression of GFP fused to the C-terminal part of histone H2B. However these nucleosomes displayed a low fluorescent intensity, not sufficient to clearly detect the autoreactive B cells (data not shown). (iii) Finally, the labeling of cysteines with AlexaFluor 488 appeared to be adapted for the study of autoreactive B cells in B6.56R mice, with a suitable fluorescence for flow cytometry use and a BCR-specific labeling.

56R mice allowed great progress in understanding the mechanisms of B cell tolerance^39, 42, 46, 55–57^. However, the study of tolerance in these mice was limited as autoreactive B cells could not be finely individualized. In fact, the description of the behaviour of IgM^a+^ cells in these animals compared to IgM^b+^ cells did not take into account the editing process of the light chain able to modify the BCR specificity. The technique described herein allowed us to identify such potentially pathogenic B cells, and could be used to further characterize the breakdown of B cell tolerance in this model, based on the anti-nucleosome activity of autoreactive B cells.

In addition, this technique could be used for the detection of autoreactive B cells in BW mice lupus nephritis models, and in SLE patients. Cysteine nucleosomes were well recognized by BW or human autoAbs, and a preliminary flow cytometric experiment showed a detectable staining on B cells from BW mice and SLE patient, although, as expected^14, 16^, the number of stained cells was low. Various improvements of this technique are still conceivable, such as the use of sorted B cells and labeled nucleosomes derived from apoptotic cells. Indeed, the immunogenicity of apoptotic nucleosomes is more important, and sera from SLE patients display an higher reactivity with apoptosis-specific chromatin modifications^58–62^. Adaptation and application of this new technique in humans would be of great interest to determine the phenotypic difference of anti-nucleosome B cells between quiescent (in inactive phase) and active SLE patients, and healthy subjects. This will undoubtedly lead to a better understanding of SLE physiopathology and to a potential effective therapeutic targeting of the pathogenic autoreactive cells, which remains a real challenge for the moment.

## Materials and Methods

### Patients and samples

5 patients with the diagnosis of SLE were selected for sera analysis. All SLE patients fulfilled the American College of Rheumatology (ACR) classification criteria for SLE^63^. Only patients with no treatment, or hydroxychloroquine, or steroids less than 20 mg per day and without immunosuppressive treatments in the previous 6 months, at the time of diagnosis, were included. The study was conducted in accordance with the principles of the Helsinki declaration. Informed consent was obtained from the parents or legal wards of the patients and the study protocol was approved by the Clinical Research Ethics Committee of Strasbourg’s University Hospital.

### Mice

47 to 51 weeks-old proteinuric female (NZBxNZW)F1 mice (also known as BW mice) or control BALB/c mice, and 7 to 9 weeks-old C57BL/6 (carrying Igh-b, i.e. the “b” allotype of heavy chain constant region) or B6.56R (carrying Igh-a, i.e. the “a” allotype of heavy chain constant region) mice were bred and maintained in the I.B.M.C. (Cellular and Molecular Biological Institute) animal facility (approved by the French Veterinary Services, #F67–482–2). B6.56R transgenic mice were genotyped by PCR amplification of tail DNA, as previously described^45^. All experiments were carried out in accordance with the European Community guidelines on the protection of animals used for scientific purposes (Directive 2010/63/UE), and approved by the Regional Ethics Committee of Strasbourg (CREMEAS).

### Nucleosome isolation

L1210 cells (murine cell line) or HEK293 cells (human cell line) were cultured in Dulbecco’s Modified Eagle’s Medium supplemented with 10% (v/v) fetal bovine serum and 100U/ml of penicillin and 100 μg/ml of streptomycin. 1 * 10^9^ L1210 cells were used to produce one batch of purified nucleosomes. Before use, 100 µL of protease inhibitor cocktail (Sigma*-*Aldrich) was added in buffers A to D. Cells were centrifuged (5 min, 400 g), then washed 2 times in 10 mL of buffer A (0.015 M TrisHCl pH 7.4, 0.015 M NaCl, 0.060 M KCl, 0.005 M MgCl_2_). After centrifugation (5 min, 400 g) the cells were washed in 10 mL of buffer B (0.01 M Tris-HCl pH 7.4, 0.001 M KCl; 0,0015 M MgCl_2_), and finally were resuspended in 10 mL of buffer B. Cells were grinded on ice for 5 minutes. 10 mL of buffer A was added prior to centrifugation (10 min, 1000 g) and the pellet was washed in 10 mL of buffer A. After centrifugation (10 min, 1000 g) the pellet was suspended in 10 mL of buffer C (0.015 M Tris-HCl pH 7.4, 0.015 M NaCl, KCl 0.060 M, 0.005 M MgCl_2_, 0.001 M CaCl_2_) and the absorbance was measured (260 nm, dilution 1:100 in 0.1% (v/v) sodium dodecyl sulfate (SDS) solution). 0.1 unit of micrococcal nuclease (Sigma*-*Aldrich) per absorbance unit was added in order to lyse free DNA. The samples were incubated 20 min at 37 °C, then 500 µL of 200 mM ethylenediamine tetraacetic acid (EDTA) was added. After centrifugation (10 min, 1500 g, +4 °C), the pellet was resuspended in 10 ml of buffer D (0.001 M Tris-HCl pH 7.4, 0.001 M EDTA pH 7.4). The solution was incubated 30 min on ice with regular shaking. After centrifugation (10 min, 1500 g, +4 °C), the supernatant – which contained the nucleosomes – was collected and dialysed (20 kDa MWCO) against 500 mL of buffer D in two successive steps at +4 °C, one for 2 hours, then overnight with fresh buffer. The DNA concentration was measured (dilution 1:100 in 0.1% (v/v) SDS solution). The samples were stored at −20 °C and referred as “native nucleosome”. All experiments were with L1210 cells derived nucleosomes, unless otherwise specified.

### Nucleosome cysteine labeling

200 µL of native nucleosome were gently mixed with 250 µL of buffer (10 mM Tris-HCl, 0.7 mM EDTA, 3.6 M NaCl). 5-fold molar excess of AlexaFluor488 C_5_-maleimide (Molecular Probes) was added and the mixture was incubated for 3 hours in the dark, at room temperature. After that, successive dialyses against solutions of decreasing ionic force was carried out following a modified protocol from Oudet P. *et al*.^33^. The solution was dialysed (3.5 kDa MWCO) in the dark at +4 °C against 500 mL of solution containing 10 mM Tris-HCl, 0.7 mM EDTA and decreasing NaCl concentration: 2 M for 2 hours, 1.5 M for 12 hours, 1 M for 24 hours, 0.75 M for 12 hours, 0.5 M for 24 hours, 0.4 M for 12 hours and a last dialysis with a new MWCO (20 kDa MWCO) against 0.4 M of NaCl for 12 hours. The DNA concentration was measured (dilution 1:100 in 0.1% (v/v) SDS solution). The samples were stored at −20 °C and referred as “cysteine nucleosome”.

### Gel characterization

200 ng of DNA from each samples were digested with 500 ng of proteinase K and was visualized after electrophoresis on 1.2% (w/v) agarose gel. For SDS-PAGE (sodium dodecyl sulfate polyacrylamide gel electrophoresis), 1.5 µg of DNA from each samples were denaturated by heating at 105 °C for 5 min in SDS reducing buffer (0.06 M Tris-HCl, pH 6.8, 2% (v/v) SDS, 5% (v/v) β-mercaptoethanol, 25% (v/v) glycerol and 0.01% (v/v) bromophenol blue). The samples were resolved using 18.0% separating gel with a 5% stacking gel. SDS-PAGE gels were then stained with Coomassie Blue.

### ELISA

96 well plates were incubated with 100 µl of nucleosome solution (DNA final concentration: 1 µg/mL) per well (L1210 cell-derived nucleosomes for mice, HEK293 cell-derived nucleosomes for humans), at 37 °C overnight. Plates were washed 3 times with PBS/0.05% (v/v) Tween (Sigma-Aldrich) and saturated with PBS/0.05% (v/v) Tween/1% (w/v) bovine serum albumine (BSA) for 1 hour at 37 °C. The plates were washed 3 times with PBS/0.05% (v/v) Tween, then 100 µL of serum (dilutions 1/500) or 100 µL of anti-H4 antibody (clone S.99.5; Thermo Fisher Scientific) were incubated for 1 hour at 37 °C. The plates were washed 3 times with PBS/0.05% (v/v) Tween, and 50 µL of an anti-human Fcγ antibody coupled to peroxidase (dilution 1/20000; polyclonal; JacksonImmuno), 50 µL of an anti-mouse Fcγ antibody coupled to peroxidase (dilution 1/10000; polyclonal; JacksonImmuno), or 50 µL of an anti-mouse Fcμ antibody coupled to peroxidase (dilution 1/10000; polyclonal; JacksonImmuno) were added, depending on the experiment. The plates were washed 3 times with PBS-/0.05% (v/v) Tween and revealed with 50 μL of TMB (3,3′,5,5′-tetramethylbenzidine), for 10 min at room temperature. The reaction was stopped with 50 µL of HCl (1 M), before reading at 450 nm using MultiScan FC (Thermo Fisher Scientific).

### Flow Cytometry

A suspension of murine splenocytes was obtained by grinding the spleen of mice on a 40 µm cell strainer. Red blood cells were lysed using ammonium-chloride-potassium lysis buffer (NH_4_Cl 0.15 M, KHCO_3_ 10.0 mM, Na_2_EDTA 0.1 mM, pH 7.4). Cells were washed twice in PBS.

Anticoagulated venous blood from patients and healthy donors was collected and was subjected to density gradient centrifugation to get peripheral blood mononuclear cells. Cells were washed twice in PBS.

Cell viability was assessed by incubation with Fixable Viability Dye eFluor 780 (eBioscience) following the manufacturer’s protocol. Cells were washed twice in PBE (PBS, 0.5% (w/v) BSA, 2 mM EDTA) and resuspended in PBE in order to have 10 * 10^6^ cells per mL. 0.5 * 10^6^ cells were dispensed into each tube. Some tubes, were incubated with 50 µL of anti-mouse IgG Fab antibodies (dilution 1/25; polyclonal; Bethyl Laboratories) for 40 min at 37 °C and washed twice with PBE. All subsequent steps are done in PBE buffer on ice, unless otherwise specified by protocol. 50 µL of labeled nucleosomes (2 µg of DNA/mL; L1210 cells derived nucleosomes for mice, HEK293 cells derived nucleosomes for humans) were added and incubated for 20 min in the dark at 4 °C. The cells were washed twice in PBE/0.1% (v/v) Tween and classical surface staining was performed in the dark for 15 min at 4 °C. When biotin conjugated antibody was used, the cells were washed twice and streptavidine was added and incubated for 15 min in the dark at 4 °C. The cells were washed twice and analysed on a Gallios flow cytometer (Beckman Coulter). A comprehensive list of antibodies used for this experiment includes: anti-mouse IgM^a^-biotin (clone DS-1), anti-mouse IgM^b^ PE (clone AF6-78), anti-mouse CD3 PerCp-Cy5.5 (clone 145-2C11), anti-mouse B220 PE-CF594 or APC (clone RA3-6B2), anti-human CD19 APC (clone HIB19), and Streptavidin APC were purchased from BD Biosciences. Data analysis was performed using Kaluza software (Beckman Coulter) with “logicle” compensation^64^. Fluorescence minus one (FMO) controls^64^ were used to make the distinction between cell populations that are positive for nucleosome staining and the ones that are negative.

### Protein sequences

Protein sequences of nucleosome core particle were defined using UniProt database (www.uniprot.org)^65^. Data used to define histone regions exposed at the surface of the nucleosome derived from previously published papers^8, 66–68^. Histone H1 (Uniprot entry: P10412-1); Histone H2A (Uniprot entry: P0C0S8); Histone H2B (Uniprot entry: P62807); Histone H3 (Uniprot entry: P68431); Histone H4 (Uniprot entry: P62805).

### Statistics

Statistical comparison between groups was carried out using nonparametric two-tailed Mann-Whitney test using Prism 5.0 (GraphPad Software Inc.). All data were presented as mean ± standard error of the mean (SEM). A p-value less than 0.05 was considered as significant.

## Electronic supplementary material

Supplementary information
