# Supplementary material for: Identification of autoreactive B cells with labeled nucleosomes
Source: Sci Rep. 2017 Apr 4;7:602. doi: 10.1038/s41598-017-00664-0 (PMC5428865; doi:10.1038/s41598-017-00664-0)
Supplement: Supplementary file 1 — Supplementary information [file 41598_2017_664_MOESM1_ESM.docx]

**SUPPLEMENTARY INFORMATION**

**Identification of autoreactive B cells with labeled nucleosomes**

Vincent Gies,^1,2^ Alain Wagner,^3^ Cécile Seifert,^1^ Aurélien Guffroy,^1,2^ Jean-D. Fauny,^1^ Anne-M. Knapp,^1^ Jean-L. Pasquali,^1,2,4^ Thierry Martin,^1,2,4^ Hélène Dumortier,^1^ Anne-S. Korganow,^1,2,4^ Pauline Soulas-Sprauel,^1,2,5^

^1^CNRS UPR 3572 "Immunopathology and Therapeutic Chemistry"/Laboratory of Excellence Medalis, Institute of Molecular and Cellular Biology (IBMC), Strasbourg, France.

^2^Department of Clinical Immunology and Internal Medicine, National Reference Center for Autoimmune Diseases, Hôpitaux Universitaires de Strasbourg, Strasbourg, France.

^3^Laboratory of of Functional ChemoSystems, CNRS-University of Strasbourg UMR 7199/Laboratory of Excellence MEDALIS, Faculté de Pharmacie, Université de Strasbourg , 74 route du Rhin, 67400 Illkirch, France.

^4^UFR Médecine, Université de Strasbourg, Strasbourg, France.

^5^UFR Sciences pharmaceutiques, Université de Strasbourg, Illkirch-Graffenstaden, France.

***Correspondence:**

Pauline SOULAS-SPRAUEL, PharmD PhD

CNRS UPR 3572 “Immunopathology and Therapeutic Chemistry”

Institute of Molecular and Cellular Biology (IBMC)

15 rue René Descartes, 67084 Strasbourg Cedex, FRANCE

Telephone number: + 33 3 88 41 70 25

Fax: + 33 3 88 61 06 80

E-mail address: [pauline.soulas@ibmc-cnrs.unistra.fr](mailto:pauline.soulas@ibmc-cnrs.unistra.fr)

**SUPPLEMENTARY MATERIALS AND METHODS**

**Fluorescence analysis**

The fluorescence of each type of nucleosome was analysed on a Gallios flow cytometer (Beckman Coulter). 5µL of each nucleosome sample was diluted in 50µL of PBS (137mM NaCl, 2.7mM KCl, 10 mM Na_2_HPO_4_, 2mM KH_2_PO_4_) and analysed on a Gallios flow cytometer (Beckman Coulter). Data analysis was performed using Kaluza software (Beckman Coulter). Fluorescence was defined as the geometric mean of fluorescence intensity (MFI).

**Immunofluorescence**

A suspension of murine splenocytes was obtained by grinding the spleen of C57BL/6 or B6.56R mice on a 40µm cell strainer. Red blood cells were lysed using ammonium-chloride-potassium lysis buffer (NH_4_Cl 0.15M, KHCO_3_ 10.0mM, Na_2_EDTA 0.1mM, pH 7.4). Cells were washed twice in PBS. Untouched B cells were sorted from splenocytes suspension using EasyStep mouse B-cell Enrichment Kit (StemCell).

Cells were washed twice in PBE (PBS, 0.5% (w/v) BSA, 2mM EDTA) and resuspended in PBE in order to have 10*10^6^ cells per mL. 0.5*10^6^ cells were dispensed into each tube. All subsequent steps are done in PBE buffer on ice, unless otherwise specified by protocol. Classical surface staining was performed with IgM-biotin (polyclonal, Jackson ImmunoResearch) in the dark for 15 min at 4°C. 50µL of labeled nucleosomes (2µg of DNA/mL) were added and incubated for 20 min in the dark at 4°C. The cells were washed twice in PBE/0.1% (v/v) Tween and streptavidine AlexaFluor 647 (BioLegend) was added and incubated for 15 min in the dark at 4°C. The cells were washed twice in PBE/0.1% (v/v). A suspension of living cells was deposited in 8 wells Nunc® Lab-Tek® Chamber Slides™ (Thermo Fisher Scientific) and were imaged using a spinning disk confocal microscope (Zeiss^®^ Axio Observer Z1, Yokogawa^®^ CSU confocal head, Zeiss^®^ Plan-Apochromat 100X/1.4 objective) or incubated with 2% paraformaldehyde in PBS (v/v) for 30 minutes. Nuclei of fixed cells were stained with DAPI (4,6 diamidino-2-phenylindole). Coverslips were mounted in Fluorescence Mounting Medium (DAKO) and sealed with nail polish. Images were acquired using a laser scanning microscope (Zeiss^®^ Axio Observer Z1, LSM 780 confocal head, Zeiss^®^ Plan-Apochromat 63X/1.4 objective). Z-stack of consecutive confocal planes were acquired. No bleed-through was detected between channels. Images were analysed using National Institutes of Health Image J software^1^.

**SUPPLEMENTARY FIGURES**

**Supplementary Figure S1.** Cysteine nucleosome production overview

**Supplementary Figure S2.** Fluorescence analysis of the nucleosomes. (**a**) Representative plots of nucleosomes analysis. (**b**) Histograms represent the MFI of native nucleosomes (*light grey*) compared to cysteine nucleosomes (*dark grey*). (**c**) The graphic represents the MFI of native and cysteine nucleosomes (mean ± SEM; n=5). Statistical comparison was carried out using nonparametric two-tailed Mann-Whitney test. Native: native nucleosome; Cysteine: cysteine nucleosome. ***P*<0.005.

**Supplementary Figure S3.** Characterization of native and labeled nucleosomes (uncropped gel). (**a**) Analysis of nucleosome DNA content (500ng of DNA) by agarose gel (1.2%) after proteinase K digestion. L: ladder; S: sample. (**b**) Analysis of nucleosome proteins (equivalent to 1.5µg of DNA) by SDS/PAGE (18%) after Coomassie Blue staining (*left*) or under UV light before Coomassie Blue staining (*right*). Native: native nucleosome; Cysteine: cysteine nucleosome; N/A: not applicable in this study.

**Supplementary Figure S4.** (**a**) Confocal microscopy Z-stack imaging of nucleosome (*green*) in IgM^+^ (*red*) sorted splenic B cells from C57BL/6 mice. DAPI (*blue*). (**b**) Immunostaining of nucleosome (*green*) in IgM^+^ (*red*) sorted splenic B cells in B6.56R mice. DAPI (*blue*). (**c**) Live imaging of sorted splenic B cells in B6.56R mice after staining with anti-IgM (*red*) and labeled nucleosome (*green*), at T = 0 min and T = 5 min, at room temperature.

**SUPPLEMENTARY REFERENCES**

1. Schneider, C. A., Rasband, W. S. & Eliceiri, K. W. NIH Image to ImageJ: 25 years of image analysis. *Nat. Methods* **9,** 671–675 (2012).
